# Personal and Social Resources Are Linked to Cognition and Health-Related Quality of Life in Childhood Cancer Survivors

**DOI:** 10.3390/children9070936

**Published:** 2022-06-22

**Authors:** Valerie Siegwart, Kirstin Schürch, Valentin Benzing, Jochen Roessler, Regula Everts

**Affiliations:** 1 Division of Pediatric Hematology and Oncology, Department of Pediatrics, Inselspital Bern University Hospital, University of Bern, 3010 Bern, Switzerland; valerie.siegwart@insel.ch (V.S.); kirstin.schuerch@insel.ch (K.S.); valentin.benzing@ispw.unibe.ch (V.B.); jochen.roessler@insel.ch (J.R.); 2 Division of Neuropediatrics, Development, and Rehabilitation, Department of Pediatrics, Inselspital Bern University Hospital, University of Bern, 3010 Bern, Switzerland; 3 Institute of Sport Science, University of Bern, 3010 Bern, Switzerland

**Keywords:** childhood cancer survivors, social and personal resources, cognitive outcome, health-related quality of life

## Abstract

Personal and social resources may buffer the adverse effects of childhood cancer and its impact on cognition and quality of life. While childhood cancer survivors show domain-specific cognitive difficulties, little is known about their personal and social resources. We therefore investigated personal and social resources and their association with cognitive and quality-of-life outcomes in childhood cancer survivors. Seventy-eight survivors of childhood cancer of different etiologies (aged 7–16 years; ≥one year since treatment) and fifty-six healthy controls were included. Cognitive outcome was assessed by neuropsychological tests; personal and social resources, as well as health-related quality of life, were assessed by standardized questionnaires. In the social resource domain, peer integration was worse in survivors than in controls (*p*_uncorr_ < 0.04, *d* = 0.33). Personal resources and all other subscales of social resources did not significantly differ between survivors and controls. In survivors, the global resource score was significantly correlated with processing speed (*r* = 0.39, *p*_corr_ < 0.001) and quality of life (parent: *r* = 0.44; self-report: *r* = 0.46; *p*_scorr_ < 0.001). In controls, no association occurred between resources and cognitive outcome, and the correlation between the global resource score and quality of life did not withstand correction for multiple comparison (parent: *r* = 0.28; self-report: *r* = 0.40, *p*_suncorr_ < 0.001). After an adverse event such as childhood cancer, resources might play a particularly buffering role on cognitive performance and quality of life (when compared to the everyday life of healthy controls). This highlights the importance of interventions that strengthen the resources of children and their families, even years after cancer. Such resource-focused intervention could help to counteract long-term sequelae in cognitive outcomes and health-related quality of life.

## 1. Introduction

Most childhood cancers share a common feature: the treatment course is long and life-threatening. Although this circumstance in general harms child development, Warner and Smith (1982) were among the first to present the idea that children might develop well despite considerable biopsychosocial risk factors [1]. Studies on resilience have led us to the conclusion that protective factors in the individual itself (personal resources) and in the environment (social resources) may help to compensate for the impact of risk factors such as cancer disease [2,3,4].

Protective resources, namely the strength and potential of patients and their families to adjust to new situations, are suggested to be the most significant determinants of a healthy adjustment to long-term stressors [5,6,7]. Protective resources are the basis for psychological health, reducing vulnerability to adverse events and strengthening resilience [7]. Protective factors help to improve resistance to risk factors and modify a person’s response to distress that in turn may lead to a maladaptive outcome [8]. It has been claimed that protective factors are identical to features of positive mental health, such as self-esteem, positive thinking, problem solving, social skills, stress management skills, and feelings of mastery [4].

In line with the view on protective resources, Hobfoll’s conservation of resources theory (1989) underlines the importance of personal and social resources as predictors of positive long-term effects [9]. Personal resources include, amongst others, optimism, a sense of coherence, and self-efficacy [10]. Social resources contain parental support, peer integration, and school integration [11,12,13]. In the literature on resources in cancer, childhood survivors showed lower self-esteem compared to healthy school children [14]. In addition, the perception of support (social resource) was associated with better social adjustment and higher psychosocial wellbeing, whereas a low sense of self-efficacy (personal resource) was associated with worse mood and worse quality of life in adult patients after cancer [15,16].

Protective resources become particularly apparent if challenged by extraordinary adversities and, consequently, should be distinguished from coping abilities used to deal with normal levels of stress in daily life [4,17]. The literature suggests a number of coping behaviors as well as categories to classify them ([18]; i.e., problem-focused adaptive coping strategies, emotion-, and appraisal-focused coping strategies). Protective resources (such as personal and social resources), however, can modify a person’s response to a distressing event. Individuals with strong protective resources are more likely to apply adaptive coping strategies, as shown in a study with adults after cancer surgery. This highlights the predictive value of resources for recovery and adjustment after cancer and the mediating role of coping [19,20]. In times of need, strong personal and social resources provide the means to deal with stress in effective ways. Previous work has noted the role of personal resources on the perception of stress in children with cancer [21,22] and distress is known to have an impact on the cognitive functions of children with cancer [23]. Hence, we assume that resources are associated with cognitive functions and health-related quality of life in particular when facing challenging life situations.

Quality of life is an all-inclusive concept that incorporates factors impacting on an individual’s life (i.e., physical, intellectual, emotional, spiritual, social, and environmental quality of life) [24]. Health-related quality of life includes only those factors that are part of an individual’s health. Non-health aspects of quality of life, such as, for example, economic and political circumstances, are not included in the measure of health-related quality of life [25]. In childhood cancer survivors, health-related quality of life was altered from the time of diagnosis onwards, throughout survivorship, with various factors, i.e., age, gender, type of cancer, treatment modality and intensity, and progression of illness shown to affect health-related quality of life [26].

Childhood cancer survivors show long-term cognitive difficulties and might present reductions in quality of life. Cognitive difficulties and reduced quality of life are linked in turn to various risk factors and are known to be worse in i.e., patients with brain tumors [27], after cranial irradiation [27] in girls [28], and in children with a young age at diagnosis [29]. Little is known about survivors’ personal and social resources and how they relate to the levels of cognition and quality of life [29,30,31]. Therefore, the present study aims to investigate the relationship between resources and cognitive outcome and health-related quality of life after childhood cancer. We hypothesize that lower personal and social resources relate to worse cognitive performance and lower health-related quality of life, in particular in childhood cancer survivors.

This study might help to explain how variability in resources potentially affects the cognitive and quality-of-life outcomes of childhood cancer survivors. An understanding of the interrelation between these domains may help the clinician to promote and develop individual therapeutic strategies that build upon strong resources while strengthening patients and their families where it is most needed.

## 2. Materials and Methods

### 2.1. Study Procedure

This cross-sectional study was part of a randomized clinical trial aiming to assess the effects of cognitive and physical training in survivors of childhood cancer (Brainfit Study) [32,33]. The study was approved by the Cantonal Ethics Committee of Bern (Bern: KEK-NR. 196/15; Zurich: ZH2015-03997), was registered at ClinicalTrials.gov (NCT02749877), and was carried out in accordance with the Declaration of Helsinki. Written informed consent was acquired from the legal guardian or from participants over 14 years of age. For the current study, we solely included baseline data; thus, none of the participants received intervention at the time of assessment.

### 2.2. Participants

The present study included 78 childhood cancer survivors (*n* = 17 with central nervous system (CNS) involvement) (for details on recruitment, see Siegwart et al., 2020) [29]. The main diagnostic categories of childhood cancer included in this study were leukemia and lymphomas (52.6%), CNS tumors (21.8%), and others (25.6%). Inclusion criteria were as follows: (a) age between 7 and 16 years, (b) diagnosed with cancer within the past 10 years (with or without CNS involvement), and (c) end of cancer treatment at least one year before participation. Further, for childhood cancer survivors without CNS involvement, cancer treatment had to comprise chemotherapy or radiation in addition to surgical removal of the tumor. Exclusion criteria were: (a) inability to follow study procedure, (b) unstable health condition, and (c) noncompliance or substance abuse.

In addition, 56 healthy controls were included. Exclusion criteria were as follows: (a) inability to follow study procedures, (b) noncompliance or substance abuse, (c) medical problems or chronic illness that can potentially influence development, and (d) mental disorders. Controls were recruited via advertisement on the hospital’s website and via public notice boards within the neighborhood. Two controls were siblings of the survivors. Healthy controls and childhood cancer survivors were comparable in terms of their age, sex, and socio-economic status (SES; see Table 1). Note that this sample represents the identical sample published in Siegwart et al., 2020 [29]).

### 2.3. Cognitive Measures

We assessed fluid intelligence using the Test of Nonverbal Intelligence [34]. Processing speed was assessed with the subtests symbol search and coding, and selective attention with the subtest cancellation of the Wechsler Intelligence Scale for Children [35]. We measured verbal memory with the subtests Atlantis and Atlantis recall, planning with the subtest Rover, and verbal working memory with the subtests word order and number recall of the German Version of the Kaufman Assessment Battery for Children [36]. Visuospatial working memory was assessed with the subtest block recall of the Working Memory Test Battery for Children [37]. Inhibition and cognitive flexibility were measured using the completion time needed for the color–word interference test of the Delis–Kaplan Executive Function System [38]. Raw scores were converted into standard scores (*M* = 100; *SD* = 15) or scaled scores (*M* = 10, *SD* = 3) based on the norms presented in the relevant test manuals. We created an executive function composite score by z-transforming each standard or scaled score (mean and standard deviation of the control group) and averaging the z-scores for each participant on the following subtests: verbal and visuospatial working memory, inhibition, cognitive flexibility, and planning.

### 2.4. Measures of Resources, Health-Related Quality of Life, and SES

To evaluate personal and social resources, the German questionnaire on resources in children and adolescents (Fragebogen zu Ressourcen im Kindes- und Jugendalter; FRKJ 8–16) was used [39]. The FRKJ 8-16 includes personal and social resource scales with six subscales for personal resources (1–6) and four subscales for social resources (7–10): (1) perspective-taking ability, (2) self-efficacy, (3) self-esteem, (4) sense of coherence, (5) optimism, (6) self-control, (7) parental support, (8) authoritative parenting style, (9) peer-group integration, and (10) school integration. Each subscale contains six items. Children’s and adolescents’ responses are measured on a scale ranging from 1 “never true” to 4 “always true”. A sample item is: “When I set my mind to something, I get it done.” The internal reliability of the subscales ranges from Cronbach’s α = 0.69 to α = 0.89 [39] and the test–-retest reliability ranges from *r* = 0.53 to *r* = 0.86 (test interval = 3 months) [39]. Raw scores were converted, based on age- and sex-specific norms, into standard scores ranging from one to nine. Higher scores indicate better resources. The respective resource scales were averaged to a global resource score based on the questionnaires’ manual. We defined a global resource score of three or less as having low resources. The questionnaire took around 20 min to be completed.

Health-related quality of life was measured using the German parent- and self-report versions of the Kidscreen-10 index [40,41]. Kidscreen-10 comprises 10 items with answers given on a 5-point Likert scale. An internal reliability of α = 0.82 and a test–retest reliability of *r* = 0.70 (test interval = 4 weeks) have been illustrated [41]. Raw scores were transformed into T-scores (mean = 50, standard deviation = 10) using existing norms for the Swiss population based on the questionnaires’ manual. Higher T-scores represent a better quality of life.

The SES was assessed using the parental net income per year and was calculated by dividing the sum of the maternal and paternal net income by two. Parents indicated their net income in a questionnaire by choosing between categories ranging from zero (no income/unemployed) to ten (>110`001 CHF/year).

### 2.5. Statistical Analyses

Statistical analyses were conducted using IBM SPSS (version 25.0; IBM, Armonk, NY, USA). We employed a multiple-imputed dataset (five imputations), employing fully conditional specification (predictive mean matching), based on all variables within the dataset [42]. Overall, 7.8% of the data points were classified as missing at random and were therefore imputed. More precisely, missing variables occurred as follows: cognitive variables: 0% to 10.4% missing; quality-of-life variables: 17.9% to 23.9% missing; personal and social resources: 22.4% missing. All of the missing data were imputed. Missing values were assignable to the following reasons: unreturned questionnaires, unavailable age norms, and non-evaluable outcome scores (e.g., due to administration difficulties). For all analyses, the alpha level was set to 0.05. Hedges’ *g* was used to estimate effect size (small effect size = 0.2, medium effect size = 0.5, large effect size = 0.8).

**Figure 1:**
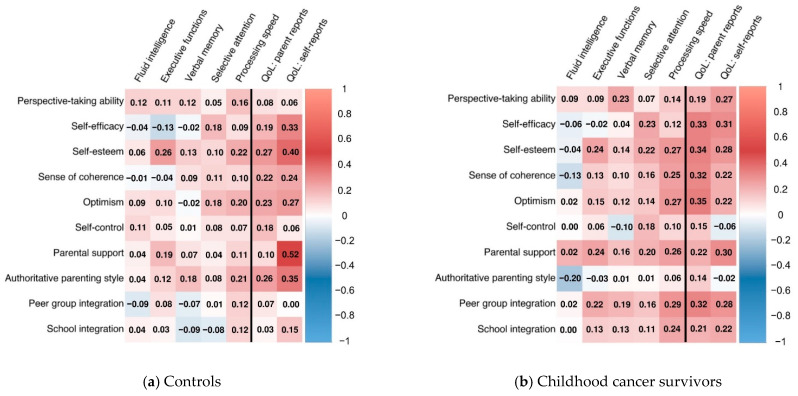
Correlational matrices between subscales of personal and social resources and cognition as well as quality of life in (**a**) controls and (**b**) childhood cancer survivors. *Note.* Two-sided Pearson correlation; color intensity indicates the strength of the correlation coefficient, red colors imply positive correlations, blue colors imply negative correlations; QoL = quality of life. No *p*-values withstand Bonferroni correction.

To compare resources between childhood cancer survivors and controls, we conducted independent-samples t-tests. Further, Pearson’s chi-square test was used to examine frequencies of low global resource scores (i.e., global resource score of ≤3). To examine the association between resources, cognition, and quality of life, we conducted Pearson’s correlation analyses in childhood cancer survivors and controls separately. The correlation coefficient ranges from −1 to +1, where a value of +1 describes a perfect positive correlation between two variables, while a correlation of −1 describes a perfect negative correlation. A correlation coefficient of zero means that there is no correlation between two variables. We compared the correlation coefficients between childhood cancer survivors and controls using a calculation in accordance with Eid et al. (2011) [43] (pp. 547 f). For data visualization, correlation matrices were generated using the R package corrplot [44]. In the correlation matrix (Figure 1), blue colors imply negative correlations, and red colors imply positive correlations. Color intensity indicates the strength of the correlation coefficient.

In addition, to compare cognitive outcome and quality of life in individuals reporting low resources (global resource score ≤ 3) with individuals reporting good resources, independent-samples t-tests were performed. We report uncorrected (*p*_uncorr_) and Bonferroni-corrected (*p*_corr_) results.

## 3. Results

### 3.1. Global Resource Score, Personal and Social Resources in Childhood Cancer Survivors and Controls

The global resource score of survivors and healthy controls was within the normative range and did not significantly differ between groups (controls: *M* = 5.45, *SD* = 1.85; survivors: *M* = 5.14, *SD* = 1.70; *t*(132) = 1.01, *p*_uncorr_ > 0.05, Hedges’ *g* = 0.18). A medium effect size indicates, however, a somewhat lower global resource score of survivors with CNS involvement (*M* = 4.47, *SD* = 1.79) than without CNS involvement (*M* = 5.32, *SD* = 1.64; *t*(132) = 1.87, *p*_uncorr_ > 0.062, Hedges’ *g* = 0.51). A low global resource score (≤3) occurred in 17% of the survivors and in 16% of the controls, a frequency that was comparable between the two groups (*χ²* = 0.008, *p*_uncorr_ > 0.561, Hedges’ *g* = 0.01).

On a subscale level, survivors showed significantly lower peer-group integration than controls (*p*_uncorr_ < 0.05, Hedges’ *g* = 0.33). All other subscales of personal and social resources were comparable between the two groups (see Table 2).

### 3.2. Associations between Resources, Cognition, and Quality of Life

In survivors, the global resource score was significantly correlated with processing speed (*r*(78) = 0.390, *p*_corr_ < 0.0005) as well as parent- and self-reported quality of life (*r*(78) > 0.440, *p*_scorr_ < 0.0005). This indicates that the survivors who reported greater personal and social resources performed better on tasks measuring processing speed and reported better quality of life (see Table 3). For the control group, none of the associations between the global resource score and cognitive or quality of life outcome were significant after Bonferroni correction. When grouping the full sample (survivors and controls) in good vs. low global resource scores (global resource score ≤ 3), individuals with low resources differed in respect to quality of life from individuals with good personal resources (*p*_scorr_ < 0.05) whereas cognitive outcome did not differ between the groups (see Table 4).

In addition, in respect to the subscales of personal and social resources, on a descriptive level the pattern of associations between processing speed and resources was stronger in childhood cancer survivors than in controls (see Figure 1). Correlation coefficients did not significantly differ between the survivors and controls (*p*_suncorr_ > 0.05).

Parental SES was unrelated to personal and social resources, to cognitive outcome, or to quality of life in survivors and controls (*p*_suncorr_ > 0.05; *r*s < 0.11). The pattern of results of the correlation analysis between personal and social resources and cognition or health-related quality of life did not change when the SES of parents was included into the analyses, neither for survivors nor for controls.

## 4. Discussion

Childhood cancer survivors did not differ from controls in personal resources such as empathy, self-efficacy, self-esteem, sense of coherence, optimism, and self-control. In addition, most of the social resources such as parental support, authoritative parenting style, and school integration did not differ between groups. The predominantly good resources in our survivor sample should be interpreted on the background of the high socio-economic status in Switzerland. The literature suggests that higher socio-economic status (measured as education, occupation, and marital status) is a protective factor for cognitive late effects in children with brain tumors [45]. However, in our study, socio-economic status was assessed as parental income, did not differ between childhood cancer survivors and controls, and was unrelated to resources, cognition, or quality of life. Hence, in our sample, financial resources are not a direct player when it comes to personal and social resources.

Despite these optimistic findings, peer-group integration was significantly lower in childhood cancer survivors than in controls. Our study shows that peer-group integration is still lower among childhood cancer survivors, even years after their last treatment. This may be related to the fact that the diagnosis and treatment of childhood cancer may lead to a decrease in the number or pattern of social contacts. Lower motor ability and physical sequelae from cancer and its treatment may hinder social activities [14]. Furthermore, support from peers may become less available due to the fear and discomfort that peers may feel toward an ill friend [46]. Our results are in consistency with findings from Dutch and Canadian studies reporting that childhood cancer survivors aged 18–30 years have fewer friends and were less likely to spend their leisure time with same-aged peers [47,48]. A disruption of social activities occurs at a time when the potential buffering effects of social support are most needed and are most likely to have an impact. The literature suggests that social support is the most important resource, in particular for adolescents and young adults with cancer [49,50,51]. When parents hold the role of primary caregivers, close relations with friends and peers are an important additional source of support and can provide emotional support during cancer and its treatment. Therefore, a loss of the feeling of being integrated within a peer group might negatively influence long-term outcomes and vice versa.

Our childhood cancer survivors with slower processing speed tend to perceive less self-esteem, less optimism, less parental support, and less peer integration (*r*s > 0.25). A study with adults with autism spectrum disorder provides additional evidence of an association between slower processing speed and impairment in social communication skills [52]. Social interactions require, among other things, rapid motor and verbal interactions among peers. In accordance with this, the slowing of response capacity in childhood cancer survivors might be a partial explanation for the worse peer integration perceived from our childhood cancer survivors.

The present results suggest a strong association between protective resources and processing speed as well as quality of life in childhood cancer survivors, an association that was much weaker in controls. Others have shown that the buffering effects of social support on, e.g., mood disturbance are best under stressful conditions [46], but may be less pronounced during periods of low stress [53]. Accordingly, resources only prove their full protective effect in the face of risks and adverse living conditions [4]. As resources are not a stable construct but can change depending on the environment (i.e., personal resources such as sense of coherence or optimism), they offer a promising opportunity to apply within preventive or therapeutic interventions. In particular, when it comes to preventive measures, the strengthening of personal and social resources should be crucial.

When interpreting our study results, some strengths and limitations have to be acknowledged. Our study is the first to combine the self and parent reports on resources and health-related quality of life with objective cognitive assessments in a rather large group of childhood cancer survivors. To investigate the interrelation of these different constructs is a holistic research approach that is rarely seen in the existing literature. As a further strength, we applied a conservative statistical approach, correcting for multiple comparisons whenever needed. Although personal and social resources can directly enhance quality of life, coping strategies have been shown to mediate the relationship between an individual’s resources and health outcomes in stressful situations [15,54,55,56]. The present study did not assess coping strategies and hence cannot replicate or refute these findings. In addition, the use of additional resource-assessing tools, such as, for example, the Social Emotional Assets and Resilience Scale (SEARS) or the Social, Cultural, Religious, Economic, Education, and Medical Family Resource Survey (SCREEM-RES), might reveal findings that add other perspectives on resources in childhood cancer survivors by the inclusion of different raters or cultural, religious, and economic issues [57,58]. Furthermore, results on personal and social resources must be interpreted in consideration of the long-term follow-up performed in the present study (mean time since treatment = 4.51 years) [14,29]. The time of the assessment of resources might impact the perception of personal and social resources. Hence, a longitudinal research approach on the development of resources and their impact on outcomes after childhood cancer should be the scope of future studies.

## 5. Conclusions

To conclude, the findings of the present study highlight that the link between personal and social resources and cognitive as well as quality of life outcome is particularly pronounced after a distressing event such as childhood cancer (when compared to healthy peers). Hence, strong resources could buffer adverse effects on cognition and health-related quality of life after childhood cancer. Furthermore, problems resulting from cancer and its treatment (i.e., problems with peer relations) could be moderated and mitigated through the strengthening of resources, and through this, an amelioration of health-related quality of life could occur [29]. Identifying available personal and social resources by means of a short questionnaire (e.g., FRKJ; [39]) or a standardized interview (e.g., focusing on the main resource domains such as optimism, self-efficacy, parental support, and peer integration) could help clinicians to determine the most effective care program in order to strengthen resources in childhood cancer survivors and their families.

## Figures and Tables

**Table 1:** Demographic and Clinical Data.

	Controls (*n* = 56)	Survivors (*n* = 78)			
	Mean (*SD*)	Mean (*SD*)	$t/\chi^{2}$	*p*	95% *CI*
	Range	Range			
Age	11.49 (2.75) 7.0–16.2	11.23 (2.49) 7.3–16.7	0.58	0.565	[−0.64–1.17]
Sex (female/male)	27/29	32/46	0.68	0.408	N/A
SES	5.54 (1.33) 2–10	5.10 (1.82) 2–10	1.56	0.122	[−0.12–1.06]
Age at diagnosis	N/A	5.38 (3.13) 0.6–12.7	N/A	N/A	N/A
Treatment duration	N/A	1.34 (0.92) 0.0–3.6	N/A	N/A	N/A
Years since cancer treatment	N/A	4.51 (2.04) 1.1–9.2	N/A	N/A	N/A

*Note.* Units of age, age at diagnosis, and treatment duration = years; N/A = not applicable; *SD* = standard deviation; *n* = sample size; SES = parental income: ranging from 0 to 10, with higher scores representing higher SES, categories ranging from zero (no income/unemployed) to ten (>110`001 CHF/year); *t* = *t*-value; $\chi^{2}$ = chi-square; *p* = level of statistical significance.

**Table 2:** Group Means of the Global Resource Score as well as all Subscales of Personal and Social Resources.

	Subscales	Controls (*n* = 56)	Survivors (*n* = 78)				
		Mean (*SD*) Range	Mean (*SD*) Range	*t*	*p*	95% *CI*	Effect Size ^b^
**Global Resource Score**		5.45 (1.85) 1–9	5.14 (1.70) 1–9	1.01	0.314	[−0.30–0.92]	0.18
**Personal resources** ^a^	Empathy and perspective taking	5.69 (2.26) 1–9	5.12 (2.30) 1–9	1.31	0.190	[−0.28–1.41]	0.22
	Self-efficacy	5.31 (2.19) 1–9	5.41 (2.31) 1–9	−0.20	0.843	[−1.17–0.97]	0.04
	Self-esteem	6.02 (2.17) 2–9	5.71 (2.01) 2–9	0.698	0.489	[−0.59–1.20]	0.15
	Sense of Coherence	5.79 (2.18) 1–9	5.27 (2.30) 1–9	1.17	0.243	[−0.36–1.39]	0.23
	Optimism	5.72 (2.18) 1–9	5.67 (1.96) 1–9	0.11	0.915	[−0.81–0.90]	0.02
	Self-control	5.75 (2.37) 2–9	5.44 (2.24) 2–9	0.69	0.491	[−0.59–1.21]	0.13
**Social resources** ^a^	Parental support	4.80 (1.62) 2–7	5.05 (1.56) 2–7	−0.88	0.377	[−0.80–0.30]	0.16
	Authoritative parenting style	5.47 (1.88) 1–9	5.75 (1.54) 3–9	−0.75	0.455	[−1.02–0.46]	0.17
	Peer group integration	5.65 (2.13) 1–9	4.91 (2.29) 1–9	2.04	0.042 *	[0.03–1.46]	0.33
	School integration	5.86 (2.02) 1–9	5.31 (1.99) 1–9	0.90	0.369	[−0.44–1.16]	0.28

*Note. SD* = standard deviation; *n* = sample size; *t* = *t*-value; *p* = level of statistical significance, * *p* < 0.05 (uncorrected for multiple comparison); ^a^ range of subscales: 1–9; 3 to 7 = average, ^b^ Hedges’ *g* for variables with unequal sample sizes.

**Table 3:** Correlations between Personal and Social Resources and Cognition or Quality of Life.

		Fluid Intelligence	Executive Functions	Verbal Memory	Selective Attention	Processing Speed	Quality of Life: Parent Reports	Quality of Life: Self-Reports
Global Resource score	Controls *n* = 56	−0.002 (0.990)	0.069 (0.613)	0.079 (0.565)	0.098 (0.477)	0.154 (0.260)	0.278 (0.038)	0.403 (0.007)
	Childhood cancer survivors *n* = 78	−0.023 (0.481)	0.223 (0.049)	0.153 (0.186)	0.220 (0.053)	0.390 (<0.0005) *	0.440 (<0.0005) *	0.460 (<0.0005) *

*Note.* Two-sided Pearson correlation, *p*-values in parenthesis. * *p-*values surviving Bonferroni correction.

**Table 4:** Group Means of Cognition and Health-Related Quality of Life in Respect to High vs. Low Global Resource Scores.

	Good Global Resource Score (*n* = 112)	Low Global Resource Score (*n* = 22)				
	Mean (*SD*) Range	Mean (*SD*) Range	*t*	*p*	95% CI	Effect Size ^a^
Fluid intelligence ^b^	105.99 (11.50) 85–132	109.45 (13.61) 82–136	−1.25	0.211	[−8.89–1.96]	0.29
Executive functions ^c^	−0.22 (0.70) −2.59–1.13	−0.22 (0.95) −3.07–0.91	0.01	0.993	[−0.34–0.34]	0.00
Verbal memory ^b^	12.17 (2.56) 8.5–17.5	11.54 (2.28) 5–17.5	1.06	0.288	[−0.53–1.80]	0.25
Selective attention ^b^	10.21 (2.96) 4.8–16	9.82 (4.35) 1–18	0.40	0.688	[−1.51–2.29]	0.12
Processing speed ^b^	104.20 (13.86) 79–134	97.68 (18.05) 53−131	1.91	0.056	[−0.16–13.19]	0.44
Quality of life: parent reports ^d^	53.29 (10.16) 31.32–77.13	44.28 (9.16) 27.9–77.13	3.56	<0.0005 *	[4.02–14.00]	0.90
Quality of life: self-report ^d^	53.00 (12.31) 28.43–81.78	42.07 (8.07) 29.03–83.56	3.86	<0.0005 *	[5.38–16.49]	0.92

*Note. SD* = standard deviation; *n* = sample size; *t* = *t*-value; *p* = level of statistical significance, * *p-*values surviving Bonferroni correction, ^a^ Hedges’ *g* for variables with unequal sample sizes, ^b^ standard or scaled scores, ^c^ composite score of EF; z-transformed values; positive values indicate better EF scores, ^d^ *T*-values (*M* = 50, *SD* = 10); higher values indicate higher quality of life.

## Data Availability

Data are available from the corresponding author upon reasonable request.
